# Unexpected Improvement of Obsessive–Compulsive Disorder Symptoms Following the Mistaken Prescription of Clomiphene: A Novel Perspective on Hormonal Influences in OCD

**DOI:** 10.1155/crps/4931083

**Published:** 2026-06-15

**Authors:** Seyed Hamzeh Hosseini, Behnam Abbasi, Fatemeh Abedian Kenari

**Affiliations:** ^1^ Psychosomatic Research Center, Mazandaran University of Medical Sciences, Sari, Iran, mazums.ac.ir; ^2^ Psychiatry and Behavioral Sciences Research Center, Addiction Institute, Mazandaran University of Medical Sciences, Sari, Iran, mazums.ac.ir; ^3^ Faculty of Medicine, Mazandaran University of Medical Sciences, Sari, Iran, mazums.ac.ir

**Keywords:** clomiphene, obsessive–compulsive disorder, unexpected response

## Abstract

**Background:**

Obsessive–compulsive disorder (OCD) is a chronic psychiatric disorder commonly treated with selective serotonin reuptake inhibitors (SSRIs) and, in more severe or resistant cases, clomipramine. Clomiphene citrate, a selective estrogen receptor (ER) modulator primarily used for infertility, is not indicated for psychiatric disorders but may influence neuroendocrine and serotonergic pathways. This case describes an unexpected improvement in OCD symptoms following the accidental prescription of clomiphene instead of clomipramine.

**Case Presentation:**

A 50‐year‐old woman with a longstanding history of anxiety and recurrent major depressive disorder (MDD) developed OCD symptoms after childbirth during her second pregnancy. Her symptoms previously responded well to sertraline, clonazepam, and clomipramine. After ~15 years of relative stability, clomipramine was discontinued because of anticholinergic side effects, resulting in relapse of obsessive–compulsive symptoms. A 3‐month trial of sertraline 200 mg/day was ineffective. When clomipramine was intended to be restarted, a prescribing error led to clomiphene 25 mg/day being dispensed instead. The patient took clomiphene together with sertraline 100 mg/day and clonazepam 2 mg/night for 12 weeks. During this period, she experienced a dramatic and complete remission of her obsessive–compulsive symptoms, with an estimated reduction in Yale‐Brown Obsessive Compulsive Scale (Y‐BOCS) score from 23 to ~7. No adverse effects were identified. After the error was recognized, clomiphene was discontinued, the incident was reported, and clomipramine was reintroduced, although the same degree of improvement was not reproduced.

**Conclusion:**

This case raises a speculative but clinically important hypothesis regarding the possible role of hormonal modulation in a subgroup of hormonally sensitive patients with OCD. However, causality cannot be established because hormonal measurements were unavailable and alternative explanations, including placebo effects, spontaneous symptom fluctuation, and concomitant medication effects, remain possible. Future studies should longitudinally assess OCD symptom changes during hormonally sensitive periods, ideally in conjunction with standardized clinical measures and hormonal biomarkers. The case also underscores the ethical and patient‐safety importance of preventing medication errors.

## 1. Background

Obsessive–compulsive disorder (OCD) is a mental health condition characterized by the presence of obsessions and compulsions. Obsessions refer to intrusive, unwanted, persistent thoughts, urges, or images that provoke significant distress. Compulsions are repetitive behaviors or mental acts performed to reduce anxiety or prevent a feared event. Overall, both obsessions and compulsions are time‐consuming, distressing, and often difficult to control or reduce despite efforts to do so [1].

The treatment of OCD has evolved over time, and selective serotonin reuptake inhibitors (SSRIs) are now considered first‐line pharmacotherapy. Clomipramine is a tricyclic antidepressant (TCA) that acts similarly to SSRIs by inhibiting the reuptake of serotonin. Because SSRIs generally have a more favorable side effect profile, they are preferred as first‐line agents, whereas clomipramine is typically reserved for more severe cases or for patients who do not respond adequately to SSRIs [2].

Clomiphene citrate, a selective estrogen receptor (ER) modulator, is typically used for female infertility. Broader neuroendocrine evidence indicates that ERs are widely distributed throughout the brain, including regions involved in affect regulation such as the hypothalamus, amygdala, hippocampus, and prefrontal cortex. These receptors influence neuronal function through genomic mechanisms, including binding to estrogen response elements and modulation of gene transcription, as well as through interactions with intracellular signaling pathways. Importantly, estrogens exert significant effects on the serotonergic system at multiple levels. Experimental studies have demonstrated that estrogen can modulate the expression of tryptophan hydroxylase (TPH), the rate‐limiting enzyme in serotonin synthesis, as well as alter serotonin transporter (SERT) expression and serotonin receptor activity, including 5‐HT1A receptors. Through these mechanisms, estrogen may influence serotonin synthesis, reuptake, and receptor sensitivity, thereby affecting mood‐related neural processes [3].

Given this interaction between estrogen signaling and serotonergic neurotransmission, it is biologically plausible that pharmacological modulation of ERs—such as through clomiphene citrate—may indirectly influence serotonergic activity in brain circuits involved in mood and behavior. Clomiphene acts as a selective ER modulator and may alter downstream transcriptional processes linked to serotonin synthesis, although direct evidence in OCD remains limited.

Although this mechanistic interpretation remains speculative, the established role of estrogen in modulating serotonergic and limbic systems provides a neurobiological framework that may help explain the observed clinical improvement in this case [3]. Psychiatric side effects such as irritability, mood swings, and feeling down have also been reported with clomiphene use [4].

In this case, clomiphene mistakenly entered into the electronic prescription system due to the similarity in the names of the drugs instead of clomipramine. After 3 months of clomiphene use, the patient showed a marked improvement in obsessive–compulsive symptoms.

## 2. Case Presentation

A 50‐year‐old woman with a longstanding history of anxiety disorders, including social anxiety beginning at age 17, presented with a complex psychiatric course. She had not sought treatment during adolescence, but at age 24, following her first childbirth, she developed acute symptoms of major depressive disorder (MDD), characterized by depressed mood, hypersomnia, reduced energy and daily activity, and poor concentration. These symptoms were accompanied by signs of anxious distress, including restlessness, a constant sense of tension, and excessive worry about something awful happening. A diagnosis of MDD with anxious distress was made according to DSM‐5 criteria. Differential diagnosis included postpartum depression because of the temporal relationship with childbirth. However, the patient fulfilled full DSM‐5 criteria for a major depressive episode. Moreover, symptom onset was not confined to the first 4 weeks after delivery, supporting a diagnosis of MDD rather than postpartum depression.

She was initiated on sertraline 100 mg daily and clonazepam 2 mg bedtime, leading to substantial symptom control. Approximately 8 years later, during her second pregnancy and following a reduction in medication doses, her depressive symptoms recurred after childbirth. At that time, in the postpartum period following her second pregnancy, she not only experienced a relapse of depressive symptoms but also developed prominent obsessive–compulsive symptoms, including contamination obsessions, cleaning compulsions, and intrusive fears of being harmed by others. According to DSM‐5 criteria, the diagnosis of MDD (Beck Depression Inventory [BDI] score 21, indicating moderate depression) and OCD (Yale‐Brown Obsessive Compulsive Scale [Y‐BOCS] score 30, indicating severe symptoms) was established [5].

At that time, treatment regimen was adjusted: sertraline was titrated back to 100 mg/day, clonazepam 2 mg at bedtime was reintroduced, and clomipramine was added and titrated up to 75 mg/day, resulting in significant improvement of depressive and obsessive–compulsive symptoms and restoration of functional status after 7 weeks with Y‐BOCS 12 and BDI 9.

After ~15 years of relative stability, during which follow‐up visits were conducted roughly every 3 months for symptom monitoring and medication management, the patient developed a resting tremor in her hands and complained of dry mouth. Considering these anticholinergic side effects and the concurrent full remission of symptoms, a decision was made to gradually discontinue clomipramine over a 4‐month period; however, this led to the recurrence of both anxiety and obsessive–compulsive symptoms consistent with relapse rather than withdrawal. An attempt was made to titrate sertraline to 200 mg/day over a 12‐week period, which the patient tolerated well without notable side effects. Despite an adequate dose and duration, this SSRI optimization phase did not result in meaningful clinical improvement, and the Y‐BOCS score remained 23 after 12 weeks. Consequently, a decision was made to reinitiate clomipramine therapy. However, due to a prescribing error in the electronic prescription system, the patient was mistakenly prescribed clomiphene 25 mg daily. She took clomiphene alongside sertraline 100 mg daily and clonazepam 2 mg nightly for 12 weeks.

Unexpectedly, during this 12‐week period, the patient experienced a dramatic and complete remission of her obsessive–compulsive symptoms, reporting high satisfaction with her treatment and resuming social and occupational activities. The patient retrospectively estimated her symptom severity as corresponding to a Y‐BOCS score of ~7; however, this estimate was based on patient recall and was not derived from a formal clinician‐administered assessment. Upon discovering the medication error during a follow‐up visit, the patient was promptly informed about the mistake in full transparency. The clinical team immediately assessed her for any potential adverse effects or complications related to clomiphene exposure and found none. The incident was documented in the patient’s medical record and reported to the institutional quality and safety committee. Clomiphene was gradually discontinued, and clomipramine was titrated to 75 mg daily. Although symptom control was eventually re‐established, the patient did not achieve the same degree of improvement that she had experienced during the clomiphene therapy. A summary of the patient’s clinical course, medication adjustments, and corresponding symptom changes is presented in Table 1.

**Table 1:** Summary of the patient’s psychiatric history, treatment timeline, and symptom progression across different medication phases.

Period/age	Key diagnosis or event	Main medications (dose)	Clinical status/Y‐BOCS	Notes
17	Onset of social anxiety	–	–	Untreated
24 (postpartum 1)	Major depressive episode	Sertraline 100 mg + clonazepam 2 mg	–	Good response
32 (postpartum 2)	Onset of OCD	Sertraline 100 mg + clomipramine 75 mg + clonazepam 2 mg	Y‐BOCS 30 → 12	Marked improvement
~47	Tremor → gradual clomipramine taper (4 months)	Sertraline ↑ to 200 mg	N/A	No improvement
48	Medication error: Clomiphene 25 mg daily	Sertraline 100 mg + clonazepam 2 mg	Y‐BOCS < 8 (retrospectively estimated)	Complete remission
49	Clomiphene discontinued; clomipramine restarted	Clomipramine 75 mg Sertraline 100 mg + clonazepam 2 mg	Y‐BOCS ≈ 12	Stable partial response

Clinical assessment in the current phase was conducted during outpatient psychiatric visits using DSM‐5 diagnostic criteria. Symptom severity was evaluated using standardized instruments, including the Y‐BOCS and the BDI administered by a trained psychiatrist.

Information regarding earlier phases of the illness, including symptom onset following childbirth, was based on retrospective patient history, as no standardized psychometric assessments had been performed at that time.

## 3. Discussion

In the presented case, a patient diagnosed with OCD, who did not respond to 3‐month course of SSRI treatment, was mistakenly prescribed clomiphene. Remarkably, after taking clomiphene for 12 weeks, the patient showed significant improvement in OCD symptoms. This observation raises a hypothesis—albeit speculative—that clomiphene, through its effects on neurotransmitter and hormonal systems, might contribute to the improvement of obsessive–compulsive symptoms. However, this assumption remains theoretical due to the absence of direct hormonal data in this case.

An important aspect of this case is the apparent relationship between reproductive hormonal events and the course of obsessive–compulsive symptoms. In this patient, clinically significant obsessive–compulsive symptoms emerged following childbirth, and symptom worsening later occurred around the age of 47, a period that may correspond to the perimenopausal transition. This temporal pattern is noteworthy, as previous research has shown that the onset or exacerbation of OCD symptoms can be associated with reproductive events, particularly during the postpartum period [6]. Additionally, evidence from systematic reviews indicates that symptoms of OCD and related anxiety disorders may fluctuate across the menstrual cycle, with exacerbations commonly observed during the premenstrual and early follicular phases, when ovarian hormone levels are declining or relatively low [7]. Importantly, such hormonal influences appear to affect only a subset of women, suggesting the existence of a hormonally sensitive subgroup. In this context, the present case may represent a hormonally sensitive form of OCD, in which modulation of the hypothalamic–pituitary–gonadal (HPG) axis could have influenced symptom expression. While this interpretation remains speculative, it may help explain the marked clinical improvement observed during clomiphene exposure.

Das et al. [8] in 2022 and Sinha and Garg [9] in 2014, reported cases of clomiphene‐induced mania, suggesting that the drug can alter mood‐related neuroendocrine pathways through testosterone modulation. Although these cases describe adverse psychiatric outcomes rather than therapeutic effects, they indirectly demonstrate that clomiphene can impact central nervous system circuits involved in affect and behavior mechanisms that could theoretically intersect with those implicated in OCD.

Interestingly, previous research has investigated the potential role of antiandrogen agents in the treatment of OCD. A systematic review by Nomani et al. [10] identified a limited number of clinical studies, including one randomized controlled trial and several nonrandomized studies, evaluating different antiandrogen agents such as flutamide, finasteride, cyproterone acetate, and triptorelin. While some of these studies reported partial improvement in obsessive–compulsive symptoms, the findings were heterogeneous and based on small sample sizes. Importantly, the overall quality of evidence was considered low, and the authors emphasized the need for further well‐designed clinical trials. These findings suggest that although androgen‐related pathways may influence OCD symptomatology, current evidence remains insufficient to support definitive therapeutic conclusions.

Recent evidence from neuroendocrinological studies has demonstrated that gonadal hormones, particularly testosterone, exert modulatory effects on key brain regions involved in social and motivated behaviors, such as the medial amygdala, bed nucleus of the stria terminalis (BNST), and medial preoptic area (MPOA). These areas express high densities of sex hormone receptors and are responsive to hormonal signaling, which can induce synaptic remodeling, alter neuronal excitability, and influence the balance of excitatory and inhibitory inputs [11]. Although this body of research primarily focuses on sexual behavior, the regulatory effects of testosterone on these limbic structures—especially the extended amygdala—support the hypothesis that testosterone may broadly influence circuits implicated in affective and compulsive behaviors.

Another study investigating neurosteroid profiles in patients with OCD demonstrated significant alterations in hormonal levels compared with healthy controls. In particular, serum concentrations of dehydroepiandrosterone (DHEA) and cortisol were found to be elevated in patients, while testosterone levels were decreased in male patients [12]. Considering that clomiphene citrate acts on the HPG axis by modulating gonadotropin release and increasing testosterone levels, it may theoretically impact the neuroendocrine imbalances observed in OCD.

The possibility of hormonal imbalances in patients diagnosed with OCD, including increased levels of testosterone, cortisol, and DHEA. DHEA may also exert bidirectional effects on GABA‐A receptors [12], which are dysregulated in OCD [13]. Broader neuroendocrine research highlights that ER signaling significantly regulates serotonin, dopamine, and glutamate neurotransmission, pathways implicated in mood regulation and obsessive–compulsive symptomatology [14]. Therefore, although speculative in a single case report, it is biologically plausible that clomiphene’s antiestrogenic action may have modified serotonergic neurotransmission, contributing to the clinical improvement observed.

Clomiphene affects several neurotransmitter systems in the brain. It competes with estradiol to bind to ERs, which are located in key regions involved in mood regulation, including the hypothalamus, amygdala, hippocampus, and cortex. The activation of these receptors influences neurotransmitter systems such as serotonin, which is primarily regulated in the hypothalamus, hippocampus, thalamus, and amygdala. Clomiphene’s antagonistic effect on ERs might disrupt serotonin synthesis by interfering with the transcriptional activity of TPH‐2, the enzyme responsible for serotonin production [15]. Serotonin dysfunction, especially through serotonin 1B and 1D receptors, has been linked to the exacerbation of OCD symptoms [16].

One possible explanation for the observed improvement is the placebo effect. Because the patient believed she was restarting clomipramine, her strong treatment expectancy might have contributed to symptom relief independent of any pharmacological action of clomiphene. The presence of significant placebo responses in OCD has been well documented in prior studies [17]. Additionally, spontaneous symptom fluctuation or regression to the mean cannot be excluded, as OCD symptoms often vary over time. These alternative explanations should be considered when interpreting the observed remission, underscoring the need for controlled studies before attributing causality to hormonal mechanisms.

It is also important to consider possible pharmacological interactions. During the clomiphene treatment period, the patient continued sertraline (100 mg/day) and clonazepam (2 mg/night). The observed improvement might have resulted from a synergistic or modulatory interaction between clomiphene and these ongoing medications rather than from clomiphene alone. Clomiphene’s potential influence on serotonergic and GABAergic systems could have indirectly enhanced the effects of sertraline or clonazepam [13]. This adjustment might have changed how the ongoing SSRI and benzodiazepine treatment affected the patient’s symptoms, though this remains a theoretical interpretation requiring further study.

Another consideration is the timing of symptom improvement. The remission became evident approximately 3 months after reintroducing pharmacotherapy, which partially overlaps with the expected response period for sertraline. However, the patient had already been taking sertraline at a stable dose for several months without benefit prior to clomiphene initiation. The rapid and marked improvement following the addition of clomiphene, after a prolonged nonresponse to SSRI therapy, raises the possibility that clomiphene may have contributed to the observed improvement, although causality cannot be established. While this temporal association does not confirm causality, it raises the possibility that hormonal modulation played a facilitative role in accelerating or enhancing therapeutic response.

Based on the present case, a hypothetical neuroendocrine model can be proposed. Clomiphene, as a selective ER modulator, may alter HPG axis activity, leading to changes in circulating sex hormone levels, including estrogen and testosterone. These hormonal changes may, in turn, influence serotonergic neurotransmission through modulation of ER signaling pathways.

**Figure 1:**
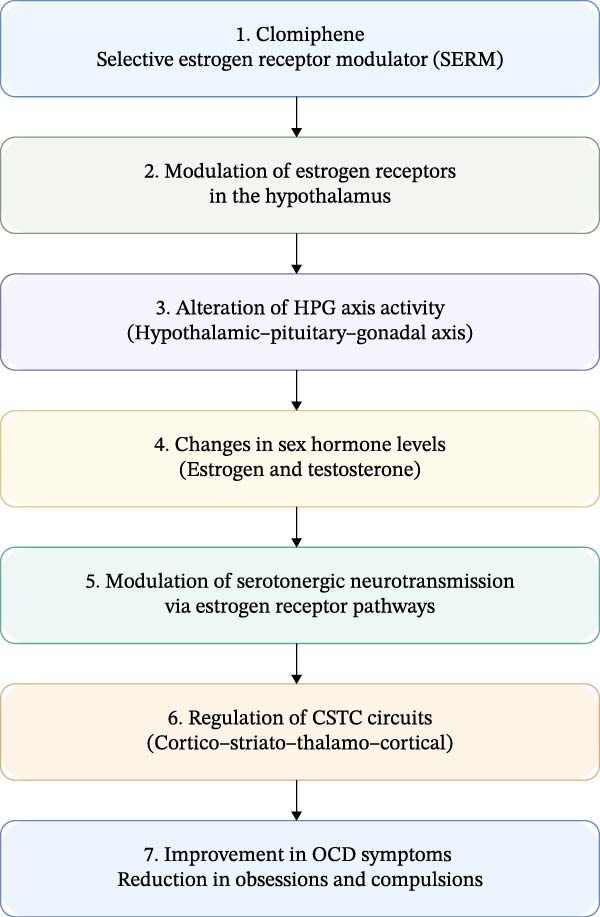
Hypothetical neuroendocrine model of clomiphene’s potential effects on OCD symptoms, illustrating modulation of the HPG axis, serotonergic neurotransmission, and CSTC circuits. The model is conceptual and intended for future investigation.

Given the established role of serotonin in cortico‐striato‐thalamo‐cortical (CSTC) circuits implicated in OCD, such neuroendocrine modulation may contribute to changes in symptom severity. This model may be particularly relevant in patients with hormonally sensitive OCD, in whom symptom expression is influenced by reproductive or endocrine factors. A proposed neuroendocrine model illustrating the potential mechanism by which clomiphene may influence OCD symptoms is presented in Figure 1.

Future studies could test this hypothesis by examining hormonal profiles and symptom changes in patients exposed to selective ER modulators under controlled conditions.

Despite the observed clinical improvement in this case, it is important to emphasize that clomiphene is not indicated for the treatment of psychiatric disorders and may be associated with adverse effects. Reported side effects include vasomotor flushing, headaches, gastrointestinal disturbances, breast tenderness, visual disturbances, and changes in mood. These effects are thought to be related to the hypoestrogenic state induced by clomiphene. Although severe adverse events are uncommon, the potential for such effects should be considered when evaluating its use [18]. Therefore, the use of clomiphene without a clear medical indication cannot be recommended.

Importantly, this case underscores a serious ethical and clinical issue. The accidental substitution of clomiphene for clomipramine represents a medication error that could have led to adverse outcomes, as the patient was unintentionally exposed to a fertility drug with potential endocrine and psychological side effects without any medical indication. Upon discovery of the error, the patient was promptly informed, and the medication was discontinued under clinical supervision. The event was reported to the institutional quality and safety committee to ensure transparency and accountability. While the unexpected improvement observed in this patient raises interesting neuroendocrine hypotheses, it is crucial to emphasize that such outcomes should never justify or minimize the seriousness of the prescribing error itself. This incident highlights the need for strict prescription verification systems, ethical responsibility in clinical practice, and organizational safeguards to prevent name‐based medication mix‐ups and ensure patient safety.

Finally, the purpose of presenting this case is not to capitalize on a clinical mistake but to highlight the importance of recognizing and learning from preventable medical errors. Reporting such incidents transparently can help raise awareness among clinicians, improve prescription safety systems, and prevent similar errors in the future. The case is shared with utmost respect for patient welfare and with the intention of contributing to safer clinical practices rather than academic gain.

### 3.1. Future Directions and Limitations

Future studies should initially focus on longitudinal observational designs rather than interventional trials. In particular, OCD symptom severity should be prospectively tracked across hormonally sensitive periods, such as pregnancy, the postpartum period, the menstrual cycle, and perimenopause, together with repeated measurements of relevant hormonal biomarkers. Such studies may help determine whether a hormonally sensitive subgroup of patients with OCD exists. A key limitation of this report is the absence of hormonal measurements before, during, and after clomiphene exposure. Therefore, any proposed mechanistic interpretation regarding testosterone, estrogen, cortisol, or DHEA alterations must be considered hypothetical and warrants future controlled investigation.

Conducting interventional studies in humans would also involve several ethical and methodological challenges. In accordance with the principles of the Declaration of Helsinki [19], any medical research involving human participants should be preceded by a careful assessment of foreseeable risks and benefits, and appropriate measures should be implemented to minimize and monitor potential harms. Because clomiphene is not indicated for psychiatric disorders and may be associated with endocrine and psychological adverse effects, any future trial would require a strong preliminary rationale, careful risk–benefit assessment, independent ethical approval, and close safety monitoring. In addition, human studies would need to control for important confounding factors. Therefore, interventional approaches should be considered only after stronger observational and biomarker‐based evidence has been established.

## 4. Conclusion

This case highlights an unexpected clinical observation that raises a speculative but important hypothesis regarding the possible role of hormonal modulation in obsessive–compulsive symptoms, particularly in a subgroup of hormonally sensitive patients with OCD. Given the temporal association between symptom changes and reproductive hormonal events in this patient, the case may support the possibility that neuroendocrine mechanisms contribute to symptom expression in selected individuals. However, no causal conclusion can be drawn from a single case, particularly in the absence of hormonal measurements and given other possible explanations, including placebo effects, spontaneous symptom fluctuation, and the influence of concomitant medications. Future research should use longitudinal and biomarker‐based approaches to clarify whether a hormonally sensitive subgroup of patients with OCD exists.

At the same time, this report underscores an important ethical and clinical point: the observed improvement occurred in the context of a prescribing error and should not be interpreted as support for the off‐label use of clomiphene in psychiatric practice. Rather, the case emphasizes the importance of medication safety, prescription accuracy, and transparent reporting of medical errors.

## Author Contributions

Seyed Hamzeh Hosseini contributed to the study conception and design and participated in the clinical management of the patient as the treating psychiatrist. Behnam Abbasi contributed to the study conception, conducted the literature review, and drafted and revised the manuscript. Fatemeh Abedian Kenari contributed to the literature review and critically reviewed and edited the final manuscript.

## Funding

No funding was provided for this study.

## Disclosure

All authors read and approved the final version of the manuscript. Treating clinicians involved in this case are among the authors; the report was prepared retrospectively and independently, with institutional ethics oversight to ensure transparency.

## Ethics Statement

Written informed consent was obtained from the patient for publication of this case report and the accompanying clinical details. All reasonable efforts were made to protect the patient’s anonymity, and no identifiable personal information is included in this report. This study was approved by the Ethics Committee of Mazandaran University of Medical Sciences under the License Number IR.MAZUMS.REC.1405.008.

## Conflicts of Interest

The authors declare no conflicts of interest.

## Data Availability

The data presented in this study are not publicly available due to ethical concerns regarding patient privacy and confidentiality. Access to these data may be granted upon reasonable request to the corresponding author, subject to approval by the relevant ethics committee.
